# Magic Cures

**Published:** 1893-01-21

**Authors:** 


### MAGIC CURES.

MANY and curious are the elements which go to make up the sum of cures by unexplained or hidden causes, popularly ascribed to magic. The stories of such cures extend from the earliest records of man down to the present day, and branch off into the domains of myth or fairy tale, religious legend and witchcraft. Certain points, amid the enormous variety of attendant circumstances in these mysterious healings, reappear with a persistency which claims special attention since they are reflected even in the most strictly scientific treatment of to-day.

Among the most prominent features of the cure by soothsayer, magician, Obeah man, and the like is the principle of a life for a life. Originally, a human life was demanded for the staying of a plague or the restoration of any illustrious sufferer. Later on it was felt that blood was the chief thing necessary, and the life of some animal was substituted. It is, of course, understood that the propitiation of some power working evil to man underlies this principle. Once admit the need for propitiating the powers of darkness, and every kind of debasing observance accumulates round it. To this principle must be ascribed all the revolting prescriptions connected with death and crime common to former ages, such as the touch of a dead man's hand for curing warts, the wearing of gallows chips for ague, the valued ingredient in potions of moss from a dead man's skull (by preference that of a thief or murderer), and all the mixed unpleasantnesses got together by Macbeth's witches.

But the sacrifice of a life was generally by the magician proper, combined with or preferred to these crooked expedients, adopted, it would seem, by the regular practitioner from a spirit of imitation, in pure ignorance of the source from whence they sprang. It may not be amiss to quote in illustration of this system of vicarious sacrifice an example taken from the life of no less a person than Blaise Pascal, and surely it was a curious bit of irony on the part of Fate to connect a "well-authenticated" story of witchcraft with the infancy of this acute thinker and severe scrutiniser of facts. The story is related by his niece.

It seems that the baby Pascal at the age of one year fell into a state of languor, varied only by extraordinary transports of rage at the sight of water, or what was still more uncanny when his father or mother drew near to each other in his presence. This went on for a year, till he was near death. An old woman was then brought to confess that she had cast a spell over the child out of spite, and that the spell was for death. "My afflicted grandfather exclaimed, 'What, must my child then die?' She replied that there was a remedy, but that someone must die in his place, and remove the spell. My grandfather said, 'I would rather my son died than that anyone should die in his stead.' She said, 'You can transfer the spell to an animal.' My grandfather offered her a horse. She told him that without going to such great expense a cat would be sufficient, and he ordered one to be given her." The cat was eventually thrown out of a window at a height of six feet from the ground, and was taken up dead. Pascal's father happily did not recollect until too late that all this involved a fresh invocation of the devil. When the true significance of it dawned on him after the child's recovery "he repented having given occasion for it." The cure was completed by the application of a poultice made of nine leaves of three kinds of herbs (three of each sort) culled before dawn by a child under seven years, and of this we shall have something to say later on.

This is a typical case. With or without the element of the previous bewitching process hundreds of cases might be cited from almost any age or country containing this central principle of a life for a life. Is it too fanciful to trace in the modern practice of vivisection a scientific development of what has been too often the mainspring of vulgar and cruel superstition? The beautiful experiment of which we read sometimes when a devoted surgeon surrenders blood from his own veins to give life to his patient marks the highest pitch to which science has climbed along a road conspicuous at its outset by its foul and crooked windings.

A second feature largely present in the recorded accounts of the magic treatment of disease is that of sympathy. Something was done to an object (generally inanimate) unconnected with the patient, or only momentarily associated with him; from this action the gravest consequences, either for good or evil, were held to ensue. The stabbing of wax effigies of their enemies, constantly charged against witches, is of this nature, and in the art of healing the principle was very widely received. A few instances must be quoted.

Boyle gives the case of a physician who, having tried all means to get rid of a "marasmus," or ague, tried the sympathetic treatment. "He took an egg and boiled it hard in his own urine; he then with a bodkin perforated the shell in many places, and buried it in a ant-hill, where it was kept to be devoured by the emmets, and as they wasted the egg he found his distemper to abate and his strength to increase, insomuch that his disease left him."

Scrofulous or ricketty children were commonly passed naked through a cleft tree. The tree was afterwards bound up and as it united the child gained strength.

A relative of the writer was strongly urged, not many years ago, by an old apple woman, to try the following cure for cataract. She was to steal a piece of beef steak, apply it for a short time to the eye, and then bury it, when the disease would pass away with the decay of the meat. A similar remedy is quoted by Grove for the cure of warts.

The eminent antiquary, Mr. Douce, says: "It is usual with many persons about Exeter who are affected with ague to visit at dead of night the nearest cross road five different times, and there bury a new-laid egg. The visit is paid about an hour before the fit is expected, and they are persuaded that with the egg they shall bury the ague."

A nail driven into an oak tree is to cure toothache, and obstinate fevers may be reduced if the patient will pare his nails, tie them to a crayfish, and throw them behind him into a river.

Such observances might be almost indefinitely multiplied. They appear to depend on a sense of the mystery of the universe; on a perception of some unseen relation of things which renders it possible at haphazard to touch springs setting dimly imagined powers at work. Something of the same blind confidence in mighty results to follow may be experienced by a princess when pressing the knob which is to launch a man-of-war. Readers of George Borrow will remember the autobiography of the man who was a victim to the mania of "touching." He relates that during an illness of his mother's he was visited in the dead of night by an irresistible conviction that if he could touch the topmost twig of a large tree near the house she would recover. At the risk of his life he performed the feat there and then, and, unhappily for the confirmation of his delusion, at the exact moment when a favourable change was noted in the invalid.

Now such beliefs had in them one element of incalculable service to mankind. They resulted in leaving the patient alone, and it may be safely concluded that any benefit accruing from the remedies above-named was due entirely to this wholesome practice. When the sympathetic system was extended to surgery and vile concoctions began to be applied, not to the wound but to the sword which had inflicted it, the innovation was wholly for good. Sir Kenelm Digby, in the reign of James I., was the first who professed to cure wounds by sympathy with the aid of vitriol p wder applied to the weapon. His cures were undeniable, but it took more than one century to trace them to their right source. Mr. Pettigrew, in his interesting account of Sir Kenelm Digby's theories, has the following remarks: "We owe to this folly the introduction of one of the first principles of surgery—one which in this country has done more to advance the science than any other beside—one which has saved a vast amount of human suffering and preserved innumerable lives. The history of the doctrine of healing wounds by the powder of sympathy is the history of adhesion—the history of union by the first intention, a history which until the time of John Hunter was never fairly developed or distinctly comprehended."

